# A modified method of lung tissue volume measurement using computed tomography numbers

**DOI:** 10.1007/s11604-025-01913-3

**Published:** 2025-11-21

**Authors:** Taiga Kobayashi, Yoshie Kunihiro, Masaki Takemitsu, Takuya Uehara, Masahiro Tanabe, Katsuyoshi Ito

**Affiliations:** 1 Department of Radiology, Yamaguchi University Graduate School of Medicine, 1-1-1 Minamikogushi, Ube, Yamaguchi 755-8505 Japan; 2 Department of Radiological Technology, Yamaguchi University Hospital, 1-1-1 Minamikogushi, Ube, Yamaguchi 755-8505 Japan

**Keywords:** Lung tissue, Lung density, Computed tomography, Anatomy

## Abstract

**Purpose:**

The values of lung tissue volume obtained by computed tomography (CT)-based analyses tend to be larger than those obtained by other methods. Establishing accurate reference values for normal lung tissue volume is important for elucidating lung changes in various diseases and may also provide reference values for information on an individual’s potential lung function and lung growth. The present study established a method for accurately calculating the lung tissue volume using CT numbers.

**Materials and methods:**

An equation for calculating lung tissue volume based on volume and composition ratio was established, and the lung tissue volume was calculated by substituting the defined CT numbers for air and lung tissue into the equation. The definition of CT number for lung tissue was determined by measuring 64 areas in 32 cases of atelectasis. The definition of CT number for air was determined by evaluating the air CT number measured in the trachea. The degree of measurement error using this method was evaluated in 12 normal cases in which CT was performed on multiple days during inspiratory and expiratory CT. The volume of lung tissue was calculated for 100 normal cases (50 consecutive males and 50 consecutive females).

**Results:**

Lung tissue volume showed a moderate correlation with height (r = 0.65, *p* < 0.0001). The strongest correlation was observed with volume of the total lung (r = 0.75, *p* < 0.0001). The lung tissue volume was approximately 530 ml for a standard body size (height, 1.7 m). The variation in the values using this method was 1.7–17.4%.

**Conclusion:**

Our study using CT numbers supports the findings of previous reports using non-image analysis. This measurement method has the potential to provide a large amount of new, noninvasive anatomical information regarding the lungs.

## Introduction

The total volume of the lungs is the same as the volume of the thoracic cavity, but it is difficult to obtain accurate information on the volume of lung tissue while excluding air. In order to evaluate the lung growth, potential lung function, changes in the structure caused by various diseases, it may be important to establish normal reference values for lung tissue volume that are not affected by volume of air.

Since 1959, when Cander et al. [1]. attempted to measure the lung tissue volume by inspiration of multiple gases, such as acetylene, there have been attempts to measure lung tissue volume using multiple methods [2–6], and in any case, it is difficult to collect large amounts of data due to their high invasiveness.

Conversely, with the development of computed tomography (CT), Gattinoni et al. devised a method for estimating lung tissue volume using CT numbers in the 1980s [7]; based on this, Cressoni et al. [8]. and Santos et al. [9]. reported measurements of lung tissue volume. This method calculates the volume of lung tissue based on the idea that the CT number of a specific pixel is a value determined by the ratio of the CT number of air ( − 1000 Hounsfield units; HU) and the CT number of lung tissue (0 HU). For example, in a pixel with a CT number of − 800 HU, it is assumed that 80% of the pixels represents air and 20% represents lung tissue. If the total lung volume was 4000 ml, and the average CT number was − 800 HU, the volume of the air would be 3200 ml and that of lung tissue would be 800 ml. This is an excellent method for obtaining large amounts of data, however, in daily clinical practice, it is empirically known that the CT number of atelectasis is close to that of soft tissue, so the CT number of lung tissue may be higher than 0 HU. In that case, the amount of lung tissue that makes up the CT number of that pixel should be smaller. In addition, although the CT number of air is theoretically − 1000 HU, in actual images, even air in the trachea does not reach − 1000 HU because of the scattering effect [10].

In the present study, cases of atelectasis observed under specific imaging conditions were collected and their CT numbers were defined as the CT numbers of lung tissue under these imaging conditions. Using this information, we devised a new formula for estimating lung tissue volume. If our method is accurate, it may provide new basic data on the anatomy of the lungs within the living body with minimal invasiveness. This could provide useful information for evaluating individual differences in lung growth and function and for comparisons with disease groups.

## Material and methods

This study was approved by the Institutional Review Board of our institution (approval number: 2024-164, Date: January 15th, 2025). The requirement for informed consent was waived because of the retrospective study design.

This study consists mainly of the following items. Construction of calculation formulas, Definition of CT numbers for lung tissue, Definition of CT numbers for air, Evaluation of measurement errors, Lung tissue volume measurements in normal cases.

### Construction of calculation formulas

1 $${\mathrm{vCT}}^{{{\mathrm{tissue}}}} + {\mathrm{vCT}}^{{{\mathrm{air}}}} = {\mathrm{vCT}}^{{{\mathrm{total}}}}$$

2 $$\begin{gathered} {\mathrm{nCT}}^{{{\mathrm{air}}}} \times {\mathrm{vCT}}^{{{\mathrm{air}}}} /{\mathrm{vCT}}^{{{\mathrm{total}}}} + {\mathrm{nCT}}^{{{\mathrm{tissue}}}} \hfill \\ \times {\mathrm{vCT}}^{{{\mathrm{tissue}}}} /{\mathrm{vCT}}^{{{\mathrm{total}}}} = {\mathrm{nCT}}^{{{\mathrm{total}}}} \hfill \\ \end{gathered}$$

In Eq. 1, the total lung volume is the sum of the air and lung tissue. In Eq. 2, the average CT number is determined by the ratio of air to lung tissue. From these two equations, we can derive the following new formula:

3 $$\begin{gathered} {\mathrm{vCT}}^{{{\mathrm{tissue}}}} \hfill \\ = {\mathrm{vCT}}^{{{\mathrm{total}}}} \times \left( {{\mathrm{nCT}}^{{{\mathrm{total}}}} - {\mathrm{nCT}}^{{{\mathrm{air}}}} } \right)/\left( {{\mathrm{nCT}}^{{{\mathrm{tissue}}}} - {\mathrm{nCT}}^{{{\mathrm{air}}}} } \right) \hfill \\ \end{gathered}$$

vCT^tissue^: volume of the lung tissue.

vCT^air^: volume of the air.

vCT^total^: volume of the total lung.

nCT^air^: CT numbers of the air.

nCT^tissue^: CT numbers of the lung tissue.

nCT^total^: average CT numbers of the total lung.

The volume of lung tissue can be calculated using Eq. 3.

### Definition of CT numbers for lung tissue

**Fig. 1:**
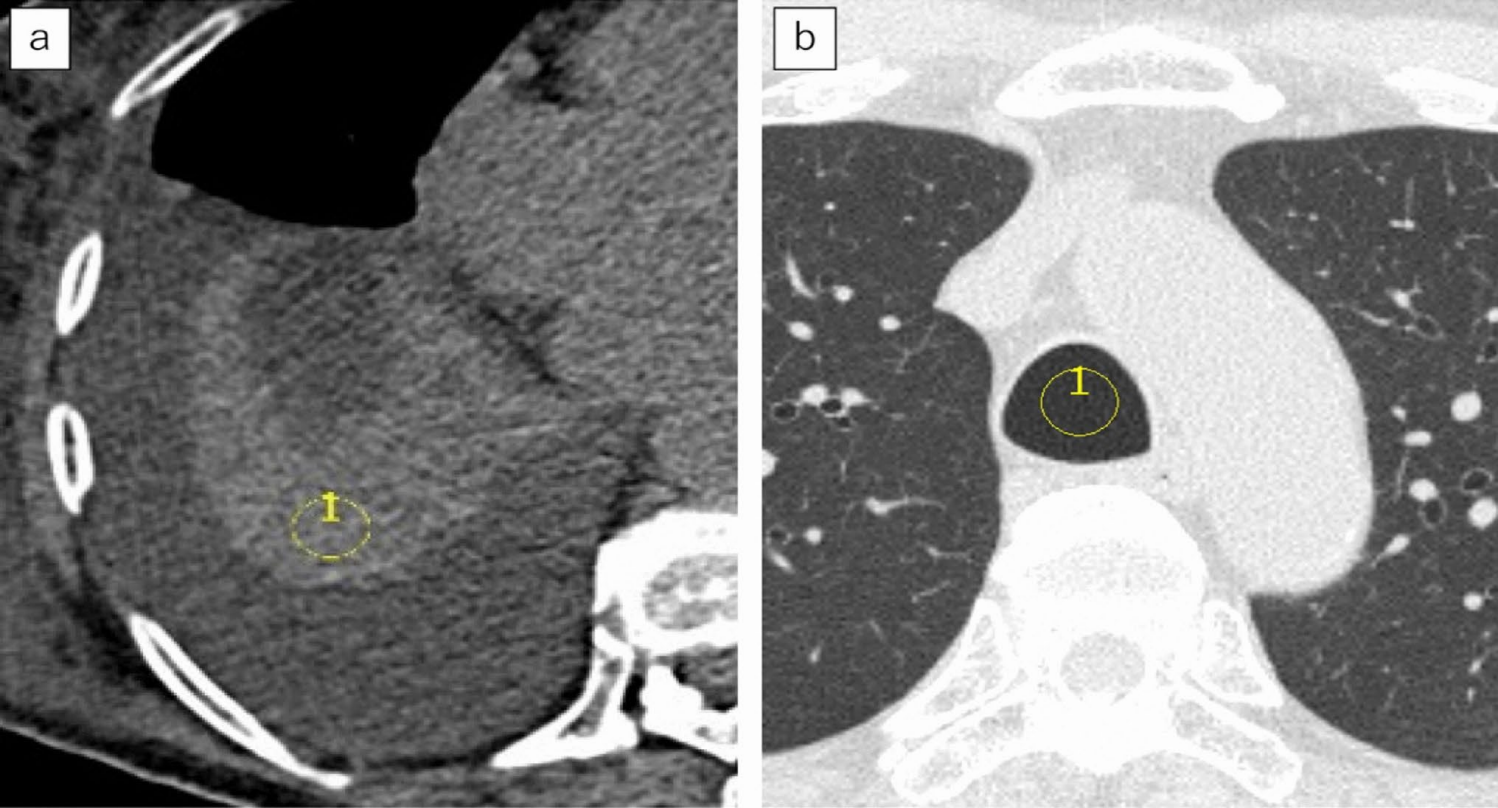
Image examples of CT number measurement in atelectasis and air in the trachea. **a** An example image of measuring CT numbers for atelectasis. A 91-year-old female. The yellow circle indicates the ROI. The central area of the atelectasis was measured, ensuring no pleural effusion or other structures were included. The left lung was similarly measured on another slice deemed optimal. **b** An example image of measuring CT numbers for air in the trachea. A 72-year-old male. The yellow circle indicates the ROI. Measurement was taken at a distance from the tracheal wall to avoid including it. *Note—*CT: computed tomography, ROI: region of interest

In order to calculate the lung tissue volume using Eq. 3, it is necessary to define the CT number of lung tissue (nCT^tissue^). Therefore, we collected cases of atelectasis and measured their CT number. We measured the average CT number of the region of interest (ROI) in the atelectasis, and defined it as the CT number of the lung tissue (nCT^tissue^) under the specific imaging conditions described in “*CT*” section. For measurement, a circular ROI with a diameter of approximately 1 to 2.5 cm was set for each case. Measurements were performed two-dimensionally on a specific axial section near the center of the atelectasis to avoid pleural effusion, normal lung fields, and bronchi (Fig. 1a). The left and right lungs were measured on separate slices determined to be optimal for each lung.

### Definition of CT numbers for air

In order to calculate the lung tissue volume using Eq. 3, it is necessary to define the CT number of air. Therefore, we confirmed whether it is more appropriate to set the CT number of air to -1000 HU or to use the CT number of air actually measured in the trachea.

**Fig. 2:**
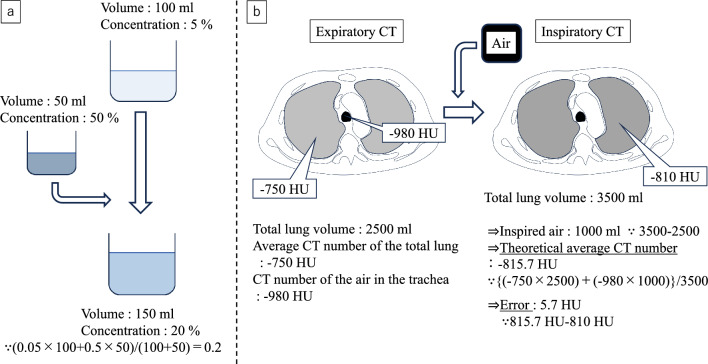
A schematic illustration of the method of calculating the theoretical average CT numbers and its error using the actual measured CT numbers of air in the trachea in expiratory CT. In the same way as the method for calculating the concentration after mixing solutions of different concentrations (**a**), the theoretical average CT numbers is calculated (**b**). If the actual average CT numbers of the total lung on inspiratory CT is -810 HU under the conditions shown in the figure, the error from the theoretical average CT numbers would be 5.7 HU. *Note*—CT: computed tomography, HU: Hounsfield units

When inspiration occurs, the total CT number of “volume of inspired air × air CT number” is diluted by the total lung volume, and the average CT number during inspiration decreases. This calculation is similar to the situation when mixing solutions with different concentrations (Fig. 2a). We decided to adopt this approach and conduct verification. The difference in volume between the inspiratory CT and expiratory CT of the lung was measured, and this value was multiplied by the CT number of air to obtain the total CT number of the inspired air. That value was added to the total CT number of the lung field in the expiratory CT, and then divided by the lung field volume in the inspiratory CT to calculate the theoretical CT number of the lung field in the inspiratory CT (Fig. 2b). We investigated the degree of error between the theoretical CT number and the actual measured CT number while changing the CT number of the air: −1000 HU, the actual measGured CT number of the air in the trachea on expiratory CT, and on inspiratory CT. The CT number of the air in the trachea was measured using the average value of a circular ROI in the trachea (Fig. 1b). We determined that the smaller the error, the more realistic the CT number of the air was and the more suitable it was for use in calculations. The calculation formula was as follows:

$$\begin{gathered} \left\{ \begin{gathered} {\mathrm{ExpnCT}}^{{{\mathrm{total}}}} \times {\mathrm{ExpvCT}}^{{{\mathrm{total}}}} + {\mathrm{nCT}}^{{{\mathrm{air}}}} \hfill \\ \times \left( {{\mathrm{InsvCT}}^{{{\mathrm{total}}}} - {\mathrm{ExpvCT}}^{{{\mathrm{total}}}} } \right) \hfill \\ \end{gathered} \right\}/{\mathrm{InsvCT}}^{{{\mathrm{total}}}} \hfill \\ = {\text{ theoretical average CT numbers}} \hfill \\ \end{gathered}$$

ExpnCT^total^: CT numbers of the total lung in expiratory CT.

ExpvCT^total^: volume of the total lung in expiratory CT.

InsvCT^total^: volume of the total lung in inspiratory CT.

### Evaluation of measurement errors

We also calculated four patterns of lung tissue volume in the same subject using inspiratory and expiratory CT data taken at different days, and compared the differences between these four values. This verification used the same case group as in *“Definition of CT numbers for air”* above.

### Lung tissue volume measurements in normal cases

Through the verification conducted in this study, we determined the CT number of the air to be used and measured lung tissue volume in normal cases. We evaluated the relationship between lung tissue volume and body size, sex, age, and total lung volume (vCT^total^), and compared the results with those reported in previous studies.

### Study subjects

#### Definition of CT numbers for lung tissue

We retrospectively collected all patients ≥ 20 years old who had bilateral atelectasis due to pleural effusion but not pneumonia or lung cancer from the CT procedures performed at our hospital between January 2023 and July 2024. From these, we extracted all cases where the extent of atelectasis was relatively large, the procedures were performed under the specific imaging conditions (i.e. 120 kVp, described in “*CT*” section).

#### Definition of CT numbers for air and evaluation of measurement error

We retrospectively collected data from patients ≥ 20 years old with normal lung imaging findings, a normal lung function, no smoking history, and multiple inspiratory and expiratory CT examinations on different days at 120 kVp within the 10-year period from January 2014 to September 2024. Cases showing nodules with calcification were excluded as potential sources of error. The solitary small nodules without calcification were considered acceptable due to their minimal volume. Mild gravity-dependent changes in the posterior peripheral lung fields were considered unlikely to significantly affect the results theoretically and were accepted as normal findings. Cases with a diagnosis of asthma or other respiratory disease were excluded, even if the images were normal. Smoking history and pulmonary function test results were extracted from the medical records. Cases with unknown smoking history were excluded. The lung image findings were confirmed to be normal based on the diagnosis by a chest radiologist with 17 years of experience (i.e. the author) and the description in the CT report. The clinical normality of the cases was determined through careful evaluation of medical records.

#### Lung tissue volume measurements in normal cases

We retrospectively collected and analyzed 100 never-smokers ≥ 20 years old with no abnormal findings in the lungs (50 consecutive males and 50 consecutive females) who underwent CT at 120 kVp between June and September 2024. In these 100 normal cases, only inspiratory CT were performed, and only inspiratory CT were used for analysis. Confirmation of normalcy was performed using a method nearly identical to that for “Definition of CT numbers for air and evaluation of measurement error”, but pulmonary function tests were not performed in these 100 cases.

##### CT

CT was performed using a normal-resolution (NR) mode with a 0.5-mm × 80 detector configuration (Aquilion Precision; Canon Medical Systems, Otawara, Japan), with a 0.6-mm × 192 detector configuration (SOMATOM Force; SIEMENS Healthineers, Erlangen, Germany), with a 0.6-mm × 64 detector configuration (SOMATOM sensation 64; SIEMENS Healthineers), with a 0.625-mm × 64 detector configuration (Optima 660 Pro, GE HealthCare, Waukesha, WI, USA), without intravenous contrast medium injection. CT images were reconstructed with a 1-mm slice thickness and a 512 × 512 matrix. The scanning parameters were 120 kVp and automated mA (except during expiratory CT with the “Aquilion Precision”, where it was fixed at 100 mA). CT images for lung and mediastinal windows were reconstructed using AIDR3D Weak FC53, AIDR3D eMILD FC03 (Aquilion Precision); ADMIRE 1 Br64, ADMIRE 2 Br40 (SOMATOM Force); FBP B70f, FBP B40f (SOMATOM Sensation 64); and ASiR 30% Lung, ASiR 30% Standard (Optima 660 Pro), respectively. The monitor utilized lung window settings (window width = 1500 HU, window level = -600 HU) and mediastinal settings (window width = 250 to 350 HU, window level = 50 HU). Only when measuring CT numbers for atelectasis were performed using mediastinal settings and all other analyses and measurements were performed using lung window settings.

The SYNAPSE VINCENT software program (version 7.0; Fujifilm, Tokyo, Japan) was used to measure the average CT numbers and volume of the lungs.

### Extraction of the measurement area

**Fig. 3:**
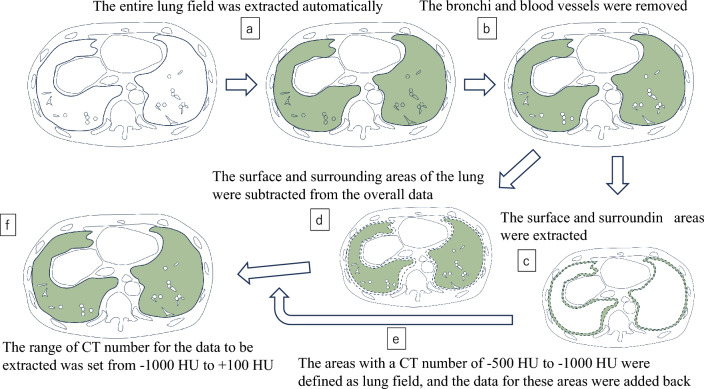
A schematic illustration of how to determine the measurement areas. The final image (**f**) is a hypothetical image calculated in this way, and in reality, this kind of image cannot be displayed. The other images (**a**, **b**, **c**, **d**) are actually displayed on the workstation. The “average CT numbers × volume” of the (**e**) is added to the “average CT numbers × volume” of the (**d**), and the final average CT numbers of the (**f**) is obtained by dividing that data by the volume of (**f**), i.e. the volume of (d + e). *Note—*CT: computed tomography, HU: Hounsfield units

Extraction of the lung fields for calculating lung tissue volume was performed as follows. First, the entire lung field was automatically extracted using the workstation functions (Fig. 3a). The areas where the bronchi, pulmonary arteries, and pulmonary veins were removed were also obtained using workstation functions (Fig. 3b). At this stage, some soft tissue may be included in the measurement target at the boundary between the lung, chest wall, diaphragm, and heart. Because the CT numbers of lung tissue are similar to those of soft tissue, including soft tissue can cause significant errors in the results, and fat can also cause errors. Therefore, the surface and surrounding areas of the lung were extracted (Fig. 3c) and subtracted (Fig. 3d) from the overall data. This extraction process could be performed automatically by the workstation’s functionality. Next, of the extracted surface and surrounding areas, only the areas with CT numbers of -500 to -1000 HU were defined as lung field, and the CT numbers and volume data for these areas were added back to the data that was previously subtracted (Fig. 3e). Furthermore, to eliminate noise in the acquired data, the CT number range of the data to be extracted was set to -1000 to + 100 HU (Fig. 3f).

### Statistical analyses

The relationship between the lung tissue volume and body size (height, body weight, body surface area (BSA)) or volume of the total lung (vCT^total^) or age were evaluated using a Spearman’s correlation analysis. An r-value > 0.7 was considered strong, 0.4 < r ≤ 0.7 moderate, 0.2 < r ≤ 0.4 weak, and r ≤ 0.2 absent. The degree of error between the theoretical and actual measured CT number while changing the CT number of the air ( − 1000 HU, measured air in the trachea on expiratory CT, and on inspiratory CT) was compared using Wilcoxon’s signed-rank test. Patient characteristics and results of lung tissue volume measurements between males and females were compared using the Mann–Whitney U test. A *p*-value < 0.05 was considered indicative of a significant difference.

All statistical analyses were performed using the JMP® Pro 18 software program (SAS Institute Inc., Cary, NC, USA).

## Results

### Definition of CT numbers for lung tissue

The collected cases of atelectasis consisted of 17 males and 15 females, with a mean age ± standard deviation (SD) of 72.9 ± 9.6 years old. We obtained measurement data for 64 areas of atelectasis in the left and right lungs. The average CT number for atelectasis was 39.5 ± 5.3 HU. This 39.5 HU was defined as the CT numbers for lung tissue (nCT^tissue^) under these imaging conditions.

### Definition of CT numbers for air and evaluation of measurement error

There were 12 cases that met the criteria within the 10-year period from January 2014 to September 2024. For each of these 12 cases, we selected the 2 with the closest examination dates. The mean number of days ± SD between two examinations was 330.75 ± 160.25 days. The error between the theoretical value and the measured value was significantly smaller when the actual measured CT numbers of the air was used, regardless of inspiration (*p* < 0.0001) or expiration (*p* < 0.0001), rather than − 1000 HU. Therefore, actual measured CT numbers were used as the CT numbers for the air in this study. When the largest of the 4 values was set to 100%, the variation in the measured values for the same case ranged from 1.7 to 17.4% (Table 1).

**Table 1:** Patient characteristics of normal lung cases with multiple inspiratory and expiratory CT scans

N*	12 (48 CT scans)
Age, years	61 ± 12
Male: female*	4: 8
Body weight, kg	56.0 ± 12.5
Height, m	1.59 ± 0.09
BSA, m^2^	1.56 ± 0.20
ExpnCT^air^, HU	−969.0 ± 17.0
InsnCT^air^, HU	− 981.6 ± 9.5
ExpvCT^total^, ml	2312.0 ± 431.7
InsvCT^total^, ml	3783.1 ± 744.7
ExpnCT^total^, HU	−754.7 ± 36.0
InsnCT^total^, HU	−835.4 ± 17.3
Theoretical CT numbers using ExpnCT^air^, HU	−838.5 ± 16.8
Theoretical CT numbers using InsnCT^air^, HU	− 843.6 ± 14.7
Theoretical CT numbers using − 1000 HU, HU	− 851.0 ± 14.6
Error between theoretical and actual CT numbers using ExpnCT^air^, HU	8.2 ± 5.6
Error between theoretical and actual CT numbers using InsnCT^air^, HU	9.7 ± 6.8
Error between theoretical and actual CT numbers using -1000 HU, HU	15.5 ± 7.0
ExpvCT^tissue^, ml	483.3 ± 81.1
InsvCT^tissue^, ml	532.1 ± 84.4
Degree of error between expirations on different days, %	8.0 ± 5.5
Degree of error between inspirations on different days, %	8.5 ± 8.1
Degree of maximum error between the four values, %	17.4 ± 5.0
Degree of minimum error between the four values, %	1.7 ± 0.9

Unless otherwise specified, values are given as the mean ± standard deviation

*Data represent the number of participants

CT: computed tomography, BSA: body surface area, HU: Hounsfield units

ExpnCT^air^: CT numbers of the air in the trachea in expiratory CT

InsnCT^air^: CT numbers of the air in the trachea in inspiratory CT

ExpvCT^total^: volume of the total lung in expiratory CT

InsvCT^total^: volume of the total lung in inspiratory CT

ExpnCT^total^: CT numbers of the total lung in expiratory CT

InsnCT^total^: CT numbers of the total lung in inspiratory CT

ExpvCT^tissue^: volume of the lung tissue in expiratory CT

InsvCT^tissue^: volume of the lung tissue in inspiratory CT

### Lung tissue volume measurements in normal cases

**Fig. 4:**
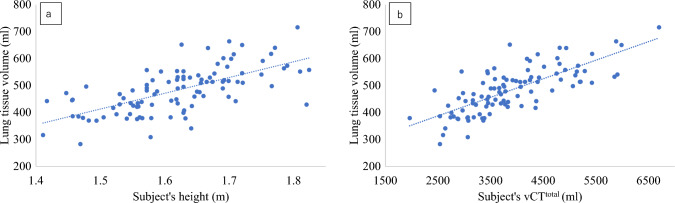
Relationship between lung tissue volume and height or vCT^total^ in 100 cases with normal lungs. **a** Height, r = 0.65, *p* < 0.0001, Lung tissue volume (ml) = 584.71 × Subject’s height (m)—464.19. **b** vCT^total^, r = 0.75, *p* < 0.0001. Lung tissue volume (ml) = 0.0689 × Subject’s vCT^total^ (ml) + 215.33. *Note—*vCT^total^: volume of the total lung

Based on the results of the previous verification, the analysis was performed using the actual measured CT number of the air instead of −1000 HU. The lung tissue volume was larger in males than in females (*p* < 0.0001), and even after adjusting for the height (*p* < 0.0001) and BSA (*p* = 0.02), a significant difference was observed (Table 2). When correlations with body size were evaluated in 100 cases, the height (r = 0.65, *p* < 0.0001), body weight (r = 0.54, *p* < 0.0001), and BSA (r = 0.61, *p* < 0.0001) were all significantly correlated, with the height showing the strongest correlation (Fig. 4a). The approximate formula for lung tissue volume when validated separately for males and females and when validated collectively for 100 cases, were as follows:

**Table 2:** Patient characteristics and result of the lung tissue volume measurement in 100 cases with normal lungs

	Population	Males	Females	*p* value
N*	100*	50*	50*	
Age, years	62.2 ± 13.8	60.5 ± 13.9	63.8 ± 13.5	0.19
Height, m	1.62 ± 0.09	1.69 ± 0.06	1.55 ± 0.07	< 0.0001
Body weight, kg	62.8 ± 14.0	70.0 ± 12.5	55.6 ± 11.5	< 0.0001
BSA, m^2^	1.66 ± 0.21	1.79 ± 0.17	1.53 ± 0.16	< 0.0001
nCT^air^ (trachea), HU	−980.9 ± 8.3	− 980.0 ± 7.8	− 981.8 ± 8.7	0.08
nCT^total^, HU	− 851.0 ± 22.3	− 855.5 ± 20.8	− 846.6 ± 23.1	0.07
vCT^total^, ml	3885 ± 909	4472 ± 827	3299 ± 546	< 0.0001
Lung tissue volume	483.0 ± 83.6	535.3 ± 64.4	430.6 ± 67.0	< 0.0001
Lung tissue volume /height, ml/m	297 ± 42	317 ± 35	277 ± 39	< 0.0001
Lung tissue volume /body weight, ml/kg	7.9 ± 1.5	7.8 ± 1.3	8.0 ± 1.7	0.90
Lung tissue volume /BSA, ml/m^2^	291 ± 40	299 ± 35	283 ± 44	0.02

Unless otherwise specified, values are given as the mean ± standard deviation

*Data represent the number of participants

*p*-values are in the assessment between males and females

CT: computed tomography, BSA: body surface area, HU: Hounsfield units

nCT^air^: CT numbers of the air

nCT^total^: CT numbers of the total lung

vCT^total^: volume of the total lung

Males: Lung tissue volume (ml) = 362.11 × Subject’s height (m) − 76.003 (r = 0.36, *p* = 0.01)

Females: Lung tissue volume (ml) = 401.49 × Subject’s height (m) − 192.33 (r = 0.39, *p* = 0.0051)

100 cases: Lung tissue volume (ml) = 584.71 × Subject’s height (m) − 464.19 (r = 0.65, *p* < 0.0001)

From the approximate formula based on height derived from the results of 100 cases, the lung tissue volume was 530 ml in a standard body-sized person (height, 1.7 m).

The volume of the total lung (vCT^total^) and lung tissue volume were strongly correlated in 100 cases (r = 0.75, *p* < 0.0001) (Fig. 4b). The respective approximation formulas using vCTtotal separated by sex, and 100 cases were as follows:

Males: Lung tissue volume (ml) = 0.0493 × Subject’s vCTtotal (ml) + 314.82 (r = 0.63, *p* < 0.0001)

Females: Lung tissue volume (ml) = 0.0666 × Subject’s vCTtotal (ml) + 210.78 (r = 0.54, *p* < 0.0001)

100 cases: Lung tissue volume (ml) = 0.0689 × Subject’s vCTtotal (ml) + 215.33 (r = 0.75, *p* < 0.0001)

The correlation between age and lung tissue volume showed no significant correlation in males (r = -0.06, *p* = 0.65). In females, there was a negative trend, but there was no significant correlation (r = − 0.27, *p* = 0.06). In 100 cases, a weak negative correlation was observed (r = − 0.21, *p* = 0.04).

## Discussion

In the present study, we derived a new formula for calculating lung tissue volume using CT numbers. The value includes the amount of pure lung tissue and capillaries, as in physiological measurement methods. According to our calculations, in the case of a standard body-sized person (height, 1.7 m), 530 ml of lung tissue contains 4035 ml of air during inspiratory CT, for a total volume of 4565 ml.

In respiratory imaging, methods using the average CT numbers of inspiratory and expiratory CT images have been shown to be highly reliable [11–15], however, our measurement method showed that even in the same subject, the value varied by 1.7–17.4%, depending on the degree of inspiration and the date of the examination. Although pulmonary vascular volume is known to be greater during inspiration than during expiration[16], all vascular structures that could be extracted were removed in our study; therefore, the effect of this macroscopic vascular volume was thought to be minimal. What is more important is the volume of pulmonary capillaries contained in the volume of lung tissue. Previous reports have estimated that pulmonary capillary volume does not change significantly between inspiration and expiration [6, 17, 18]. Therefore, the variation in the values obtained in this study is thought to be due to errors in the analysis and measurement methods used, rather than actual changes in lung tissue volume due to respiration. We suspect that this is because the noise affecting the image is not always constant and may change with each procedure depending on the position, the degree of respiration, and other factors.

The findings that males have more lung tissue than females and that there is a correlation with height agree with a previous report [8]. The values of the lung tissue volume obtained in this study are generally similar to those obtained in previous studies using physiological or morphometrical method and were smaller than those estimated from the CT numbers (Table 3). Theoretically, if we set the CT number for lung tissue as 0 HU and that of air as − 1000 HU versus calculating the CT number for lung tissue as 39.5 HU and that of air as − 980 HU, the latter measurement of the lung tissue volume will be around 80–90% of the former measurement. However, the difference in the results measured in this study was even greater, at approximately 60% of the results in previous reports [8, 9]. Considering that the average CT numbers of the lung in previous reports were higher than our measurements, this may be due to the differences in the method of region extraction. In our case, we remove soft tissue located at the boundary between the chest wall and mediastinal tissue to eliminate causes of overestimation.

**Table 3:** A comparison with previous reports on lung tissue volume or lung weight in normal subjects

	Method	N	Listed value	Standard body size conversion, ml	Standard body size conversion, g
Present study	CT numbers	100	483 ml	530 (height)	
Cressoni[8]	CT numbers	100	929 ml	971 (height)	
Santos[9]	CT numbers	8	728 ml	778 (height)	
Cander[1]	Gas inspiration	5	627 ml	582 (height)	
Kalley[19]	Gas inspiration	6	387 ml/m^2^ (BSA)	670 (BSA)	
Gonzalez[20]	Gas inspiration	11	559 ml	564 (height)	
Gehr[3]	Morphometrics	8	7.38 ml/kg (BW)	465 (BW)	
Gump[4]	Anatomy	5	325 g/m^2^ (BSA)		562 (BSA)
Hsia[6]	Morphometrics	7	300 g*		291 (height)*

*Capillary blood weight may not be included

The values obtained by converting the values given in previous reports to standard body size data (height of 1.7 m, body weight of 63 kg, and BSA of 1.73 m^2^). The criteria used for conversion are shown in parentheses

CT: computed tomography, BSA: body surface area, BW: body weight

It is difficult to compare our findings with anatomical methods for which weight is measured; however, if we assume 1 g = 1 ml in accordance with the convention, the values are similar. Although it is larger than the value estimated from the structural analysis using an electron microscope [6], if the weight of the blood in the capillaries is not included in that value, adding it may result in a closer value. Various values have been reported for capillary blood volume, ranging from 40 to 210 ml [3, 21–23], and Hsia et al. reported a capillary volume of 200 ml [6].

In the 100 cases, a weak negative correlation was observed between age and lung tissue volume. In the previous report [8], a similarly weak negative correlation was observed. Our results demonstrate reproducibility with their study and suggest a potential negative correlation with age. However, the statistical power of this study is insufficient to provide conclusive evidence. This study focused on a group of elderly individuals within a relatively narrow age range, and it is considered that verification should be conducted using a broader age range for evaluating the relationship with age. It will be necessary to add such verification in the future.

Several limitations associated with the present study warrant mention. First, this study is a retrospective study. In healthy subjects, cases where multiple inspiratory and expiratory CT scans were performed are extremely rare, and only 12 cases could be collected over a 10-year period. This suggests that the sample size is insufficient for verifying the CT numbers of air or analyzing their accuracy in this study. Furthermore, as this study is limited to a single institution and a specific racial group, so caution is warranted in immediately generalizing our findings. Second, the fact that the actual measured CT numbers of the air in the trachea is not − 1000 HU is thought to be due to the scattering effect, but it is difficult to evaluate the exact impact on our measurement results. Third, the fact that the target range for CT number analyses has been narrowed from − 1000 to 100 HU means that only noise below − 1000 HU is removed from the examination results, and values that fall within this range despite being affected by noise are considered acceptable. In addition, values below − 1000HU may also be removed even though they are not strictly noise. Fourth, because atelectasis includes large blood vessels, it cannot strictly refer to pure lung tissue. In addition, since the condition of atelectasis differs from that of normal lungs, CT numbers cannot be considered equivalent to those of completely normal lung tissue. Fifth, multiple CT models were used in this study. Theoretically, when using actual measured value, there was no marked difference in the lung tissue volume values, even if the model was different. However, there is a possibility of error because the CT numbers of the lung tissue were fixed. It is possible that this was one of the causes of small errors. Furthermore, in some imaging sessions, the tube current during expiratory CT was fixed, which may have slightly increased the impact of noise.

In conclusion, the results obtained in this study using CT numbers filled the gap between the lung tissue volume reported by non-image analysis methods and those reported by image analysis methods. Our method enables noninvasive measurement of lung tissue volume, and in the future, accumulating more cases may enable the large-scale acquisition of noninvasive data on individual lung growth and potential lung function.
